# GCN-5/PGC-1α signaling is activated and associated with metabolism in cyclin E1-driven ovarian cancer

**DOI:** 10.18632/aging.102082

**Published:** 2019-07-17

**Authors:** Ting Guo, Bin Li, Chao Gu, Xiuying Chen, Mengxin Han, Xiaocheng Liu, Congjian Xu

**Affiliations:** 1 Department of Gynecology, Obstetrics and Gynecology Hospital of Fudan University, Shanghai 200011, PR China

**Keywords:** high grade serous ovarian cancer, CCNE1, glucose metabolism

## Abstract

Aim: We previously found Cyclin E1-driven high grade serous ovarian cancer (HGSOC) showed metabolic shift. In this study, we aimed to elucidate signaling pathway therein.

Methods: In silico reproduction of TCGA ovarian cancer dataset and pathway analysis were performed. Candidate metabolic pathway was validated using *in vitro* and *in vivo* assays.

Results: We found CCNE1-amplified HGSOC showed significant metabolic alteration besides canonical cell cycle control. Using CCNE1-amplified OVCAR-3 and A2780 OvCa cells, we found that knockdown of CDK2 and GCN5 resulted in decreased G6PC and increased PGC-1α level, and that both genetic and pharmaceutical (MB-3) inhibition of GCN5 resulted in significant decrease in acetylation of PGC-1α. Silencing of CDK2 also resulted in significant decrease in acetylation of both PGC-1α and Rb. GCN5-KD significantly decreased glucose uptake, increased lactate production and decreased SDH activity. Western blots showed hierarchy of the elements indicating Cyclin E1-CDK2/GCN5/PGC-1α regulatory axis from up- to down- stream. Inhibitory effect of Dinaciclib was similar to that of GCN5 silencing, whereas combination therapy further inhibited cell proliferation significantly. Similar findings were noted also in cell cycle arrest, apoptosis, invasion, migration and colony formation assays. Xenograft experiments showed that GCN5-KD alone did not alter tumor growth yet combination therapy of Dinaciclib and GCN5-KD conferred significant inhibition of tumor growth compared with either therapy alone with no toxicity observed.

Conclusion: GCN-5/PGC-1α signaling is activated and associated with metabolism in Cyclin E1-driven HGSOC. Targeting GCN5 hold promise to augment current CDK2-targeting strategy and further studies are warranted for clinical translation.

## INTRODUCTION

Approximately 220,000 women are affected by malignant epithelial ovarian tumor each year and ovarian cancer (OvCa) is the fifth commonest cause of death in women with cancer in the United States [1]. High grade serous ovarian cancer (HGSOC) consists of 70% of OvCa cases that decease [2]. HGSOC at late stage responds poorly to platinum-based chemotherapy, which is the standard-of-care. Rapid relapse and unfavorable overall survival are critical clinical plights [3].

HGSOC is highly heterogeneous with different genetic subtypes conferring distinct prognosis. Besides well-established truncal TP53 mutations, HGSOC is also characterized with high frequency of CCNE1 amplification and resulting upregulation (~30%). This genotype is termed Cyclin E1-driven HGSOC [4]. Cyclin E1 is encoded by CCNE1 and forms complex with Cyclin Dependent Kinase 2 (CDK2). The complex plays critical role in regulation of G1/S phase and incurs in part the uncontrolled cell cycle progression in cancer context. Unlike other genotypes, Cyclin E1-driven HGSOC are insensitive to platinum-based chemotherapy and resistance surfaces even at initiation. Thus, such genotype is associated with sooner relapse and worsened prognosis [5]. Notwithstanding exploratory administration of CDK2 in Cyclin E1-driven HGSOC showing promising efficacy, future resistance remains unsolved [6].

Our previous reports indicate that Cyclin E1-driven ovarian cancer is sensitive to inhibition of both CDK2 and glucose metabolism. We also report that G6PC may stay at the hub of glucose metabolism and cell cycle mediation. Those findings shed light on novel function of Cyclin E1-CDK2 complex, which may in our speculation mediate crosstalk between cell cycle regulation and metabolism, a mechanism that may contribute to resistance to cell cycle based inhibitors like chemotherapy or CDK2 inhibition [7, 8]. In a recent study focusing on gluconeogenesis, CITED2 was reported to inhibit the acetylation of PGC-1a by blocking its interaction with the acetyltransferase general control of amino acid synthesis 5–like 2 (GCN5) and the acetylation of PGC-1a was GCN5-dependent. Together with our findings, we speculate that the GCN-5/PGC-1α may also play a role in ovarian cancer [9].

In the current study, we focus on the mechanism of resistance to CDK2 inhibition in Cyclin E1-driven HGSOC. With *in vitro* and *in vivo* modeling of the genotype, we aim at breaking bottleneck of treatment dilemma of OvCa and our findings may hold promise to precision targeted therapy for OvCa.

## RESULTS

**Figure 1:**
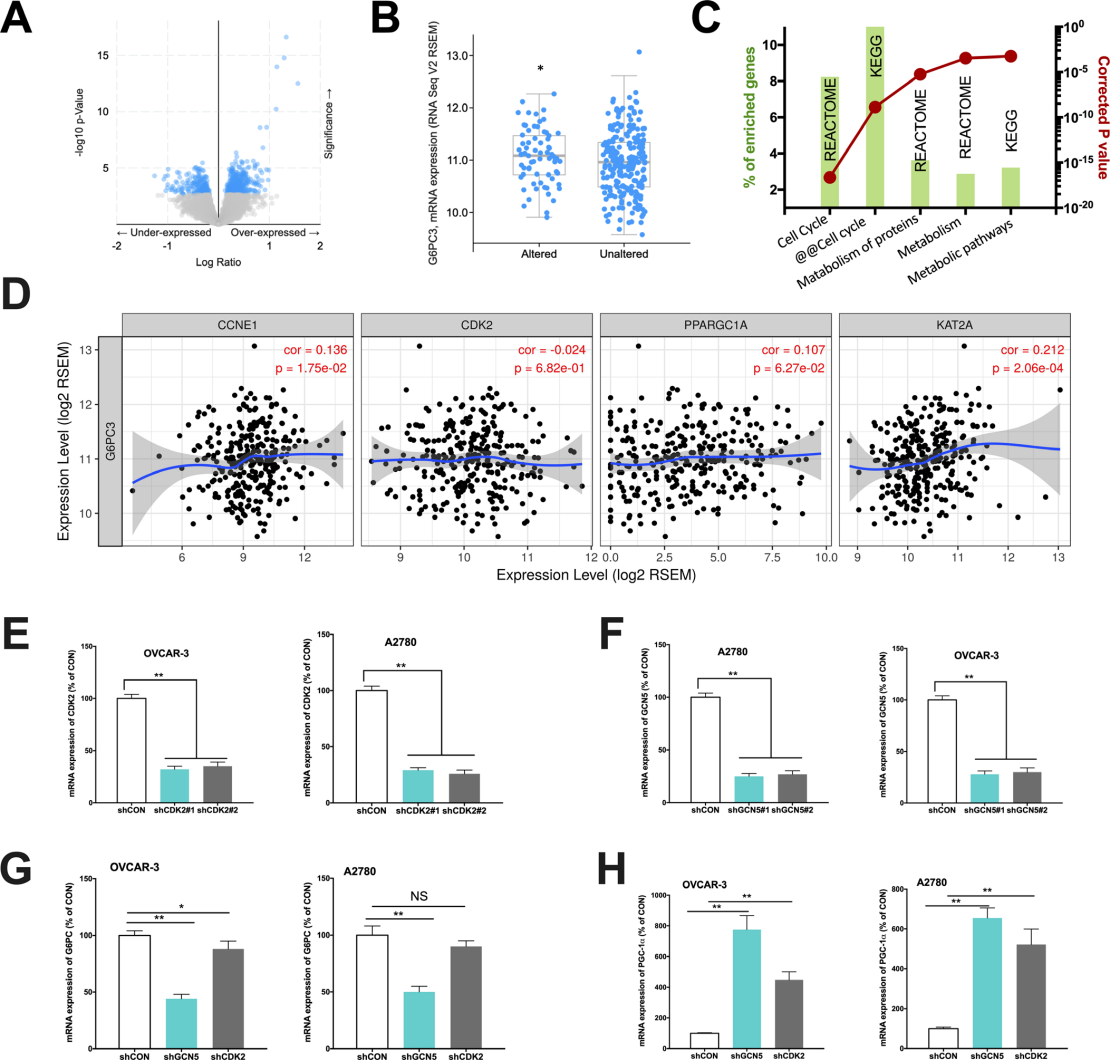
Cyclin E1-driven HGSOC shows metabolic alteration. Reproduced from TCGA, shown are (**A**) significantly enriched gene expressions (blue), amongst which included (**B**) significantly upregulated G6PC3, (**C**) functional annotation revealing significantly altered metabolic pathways and (**D**) correlations of expressions of CCNE1 and metabolic genes; In two CCNE1-amplified HGSOC cell lines, shown are silencing efficacy of (**E**) CDK2 and (**F**) GCN5; and expressions of (**G**) G6PC and (**H**) PGC-1α. (*P < 0.05; **P < 0.01)

In the current study, we first aim to validate our speculation whether CCNE1 amplified HGSOC has altered metabolic pathway via GCN5/PGC-1α/G6PC axis. We first analyzed expressions of genes enriched in CCNE1 amplified cases (~22% of cases) and found that genes significantly enriched were at abundance (Figure 1A) and that the subunit of G6PC, the G6PC3 was significantly upregulated in CCNE1 amplified cases (Figure 1B). This was not surprising as the sequencing dataset we selected only encompassed G6PC3 subunit of the gene, yet considering the functional redundancy we proceeded with *in vitro* probing. Functional annotation of enriched genes showed that except for cell cycle pathway significantly altered which was established, the second most significantly altered pathway was metabolism (Figure 1C). We then studied correlation between expressions of genes within the signaling axis using the mRNA expression data from TCGA cohort. Using partial correlation simulation, we observed significant positive correlation between CCNE1 and G6PC3 expression, indicating potential positive regulation between the two. We also noticed positive correlation between expressions of KAT2A (GCN5) and G6PC3, also indicating potential positive regulation between the genes. No correlation was noticed for CDK2 or PGC-1α with CCNE1 (Figure 1D). Here we showed in clinical tissue samples, echoing our previous work that metabolism plays a role in CCNE1 amplified OvCa and G6PC may play a role therein linking GCN5 and PGC-1α.

**Figure 2:**
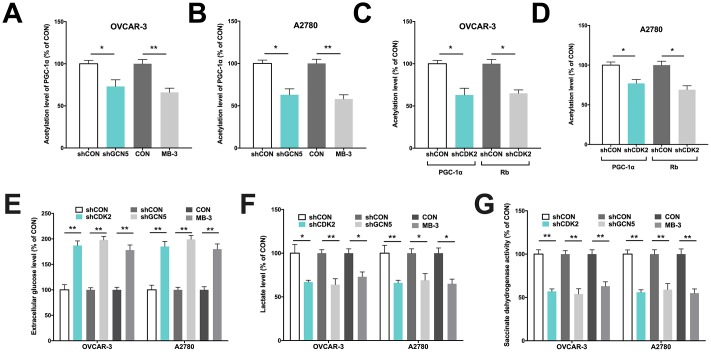
GCN5/PGC-1α axis mediates metabolism in Cyclin E1-driven HGSOC. Shown are acetylation of PGC-1α by GCN5 silencing in (**A**) OVCAR-3 and (**B**) A2780 cells, and acetylation of PGC-1α and Rb by CDK2 silencing in (**C**) OVCAR-3 and (**D**) A2780 cells; Shown are (**E**) glucose consumption, (**F**) lactate secretion, (**G**) succinate dehydrogenase activity in cells with CDK and GCN5 silencing. (*P < 0.05; **P < 0.01)

We next performed *in vitro* studies to elucidate mechanistic regulation. Both OVCAR-3 and A2780 OvCa cells were confirmed to be CCNE1 amplified. By silencing CDK2 and GCN5 in both cell lines (Figure 1E–1F), we found that KD of GCN5 resulted in decreased G6PC and increased PGC-1α level in both cell lines, respectively (Figure 1G–1H). CDK2-KD significantly lowered expression of G6PC in OVCAR-3 cells but not in A2780 cells (Figure 1G). CDK2-KD significantly increased expression of PGC-1α in both cell lines (Figure 1H). We then investigated whether negative regulation of CDK2 and GCN5 on PGC-1α was due to acetylation. We found that both genetic and pharmaceutical (MB-3) inhibition resulted in significant decrease in acetylation of PGC-1α in both cell lines (Figure 2A–2B). Silencing of CDK2 also resulted in significant decrease in acetylation of both PGC-1α and Rb (Figure 2C–2D), supporting the decreased mRNA expression of PGC-1α could be due to inhibition via acetylation by upstream CCNE1/CDK2/GCN5 signaling. We next examined metabolic profiles and found silencing of GCN5 genetically or pharmaceutically had similar effect to CDK2-KD by significantly decreasing glucose uptake (Figure 2E), increasing lactate production (Figure 2F), and decreasing SDH activity (Figure 2G).

**Figure 3:**
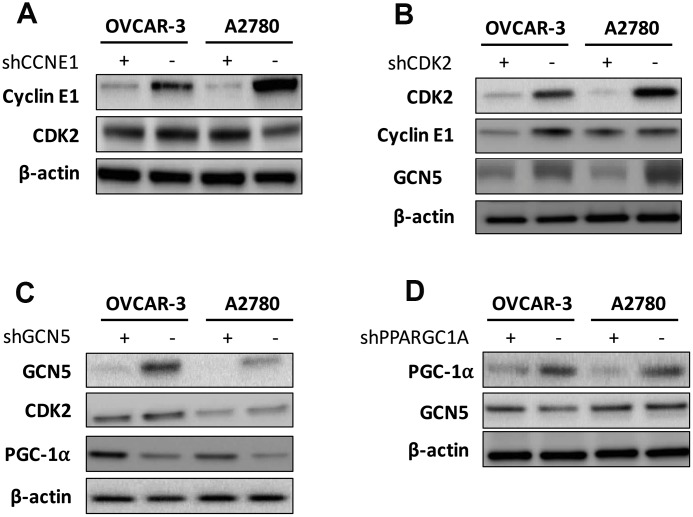
Shown are hierarchy of regulation demonstrating impact of (**A**) silencing of CCNE1 on CDK2; (**B**) silencing of CDK2 on Cyclin E1 and GCN5; (**C**) silencing of GNG5 on CDK2 and PGC-1α; and (**D**) silencing of PGC-1α onGCN5.

We next tried to identify hierarchy of the elements. We first found that silencing of CCNE1 had minute impact on CDK2 level (Figure 3A), in reminiscence of a recent report that overexpression and amplification of CCNE1 is dependent on CDK2 activity in Cyclin E1 driven cancer cell lines. We then silenced CDK2 in both cell lines and found a corresponding decrease in CCNE1 level in OVCAR-3 cells but not as apparent in A2780 cells, indicating individualized mechanism in crosstalk between CCNE1 and CDK2 (Figure 3B). Also, CDK2 silencing resulted in decreased GCN5 level in both cell lines (Figure 3B). Silencing of GCN5 showed minimal impact on CDK2 level yet resulted in increased PGC-1α level in both cell lines (Figure 3C). Silencing of PGC-1α also showed minimal impact on GCN5 level (Figure 3D). We thus speculate that the Cyclin E1-CDK2 complex mediates GCN5 which subsequently inhibits PGC-1α by acetylation.

**Figure 4:**
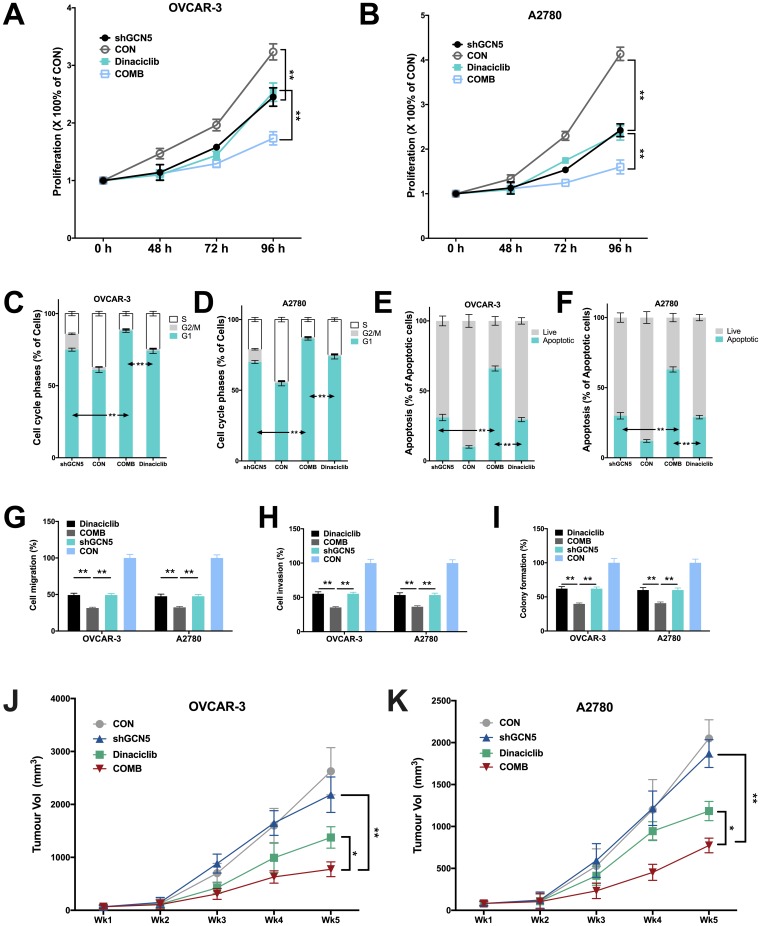
**Combination therapy of targeting GCN5 and CDK2 inhibits CCNE1-amplified HGSOC.** Shown are inhibitory effects of shGCN5 and CDK2 inhibitor Dinaciclib in proliferation assay in (**A**) OVCAR-3 and (**B**) A2780 cells; combination therapy in cell cycle assay in (**C**) OVCAR-3 and (**D**) A2780 cells; combination therapy in apoptosis assay in (**E**) OVCAR-3 and (**F**) A2780 cells; combination therapy in Transwell (**G**) migration assay and (**H**) invasion assay, and in (**I**) colony formation assay; (**J**, **K**) Xenograft mouse model showing tumor inhibitory effects using genetic silencing of GCN5 and CDK2 inhibitor. (*P < 0.05; **P < 0.01)

We next studied whether inhibiting GCN5 could have additive effect to CDK2 inhibitor. As the Cyclin E1-CDK2 complex plays pro-tumorigenic role in a variety of mechanisms, inhibition of GCN5 is not expected to substitute CDK2 inhibition despite its downstream position in the signaling. In both cell lines, we found that inhibitory effect of Dinaciclib was similar to that of GCN5 silencing, whereas combination therapy further inhibited cell proliferation significantly (Figure 4A–4B). The cell cycle analyses revealed that both Dinaciclib and GCN5-KD resulted in increased G1 population in both cell lines, whereas Dinaciclib almost completely blocked cell entering G2 phase (Figure 4C–4D). Combination therapy augmented G1 phase population leaving only minimal population in G2/M phase (Figure 4C–4D). Combination therapy also significantly increased cell apoptosis as compared to either blockade in both cell lines (Figure 4E–4F). We next studied inhibitory effect on migratory ability of cells and found that combination therapy lead to significantly additive inhibition in cell migration and invasion as compared to GCN5-KD or Dinaciclib alone (Figure 4G–4H). Combination therapy also conferred significantly additive inhibition to anchorage-independent growth in both cell lines (Figure 4I). Finally, we performed xenograft experiments and found, intriguingly, GCN5-KD did not alter tumor growth compared with control in both cell lines (Figure 4J–4K). However, combination therapy of Dinaciclib and GCN5-KD conferred significant inhibition of tumor growth compared with either therapy alone (Figure 4J–4K). No toxicity was observed in mice throughout the experiment period.

## DISCUSSION

In the current study, we have provided evidence that CCNE1 drives metabolic alteration in ovarian carcinoma with activated GCN5/PGC-1α signaling. The hypothesis of this study was proposed based on 2 previous studies by our group showing unique metabolic shift in ovarian cancer [7, 8]. We first discovered association between G6PC and CDKN1B in HGSOC and that Warburg effect was not the prominent metabolic shift in HGSOC. In our later study, we showed that Cyclin E1-dirven HGSOC was dependent on tricarboxylic acid cycle upon CDK2 inhibition, further supporting a connection between cell cycle control and glucose metabolism in this genotype. We thus proposed that Cyclin E1-CDK2 complex may stand at the hub of mediating both cycle and metabolism, and while the mechanism of former has been well studied, we here focused on its signaling in glucose metabolism.

Crosstalk between cell cycle and glucose metabolism plays critical role yet has not been well elucidated. Cyclins are a series cell cycle-dependent proteins controlling checkpoint passage via their activity and quantity. The dynamic change in Cyclin amount ensures no excessive proliferation and adequate time for DNA repair under normal circumstances [10]. Cyclin E1 belongs to cyclin family and binds to CKD2 as a compound which phosphorylates downstream proteins, playing an important role in G1 phase and G1-S transition of mammalian cell cycle progression. Due to the amplification of genes, Cyclin E1 RNA is overexpressed in 29.5% and CKD2 in 6.5% of ovarian tumors tested, making it another hallmark of ovarian cancer besides BRCAness [11]. So far metabolic mediation of Cylcin E1-CDK2 has not been reported besides our group. However, similar mechanistic analyses have shown that Cyclin D1-CDK4 complex can regulate glucose metabolism in hepatic cells via cell cycle-independent pathways [12]. Also, loss of cell cycle controlling gene CDKN2A, another commonly deleted gene in cancer, can break glucose equilibrium and cause increased insulin secretion by pancreatic β cells [13]. We therefore postulate that the signaling between Cylcin E1-CDK2 and downstream G6PC is via GCN5/PGC-1α axis, which has been established to mediate glucose metabolism [9].

By elucidating in part the signaling, we also evaluated the therapeutic potential of GCN5 inhibition alone and in combination with CDK2 inhibitor. Genetic inhibition of GCN5 has been shown to suppress human hepatocellular carcinoma and leukemia [14, 15]. However, the substrates reported to be acetylated are different by GCN5 in those reports. In the current study, we show that GCN5 acetylates PGC-1α and leads to alteration of glucose metabolism. Though we revealed in part the interaction between GCN5 and PGC-1α, Co-IP is now being performed to validate the findings. We also noted that although pharmaceutical GCN5 inhibition shows potent effect alone or in combination with CDK2 inhibition, it is currently unsuitable for *in vivo* use as the toxicity was strong (data not shown) and that’s the reason we used genetic silencing in animal models for xenograft tumor. While silencing GCN5 shows effect, reducing toxicity in pharmaceutical development remains challenging. We also noticed that genetic silencing of GCN5 alone showed only showed inhibitory effect *in vitro* but not *in vivo*. We speculate that the complexity of microenvironment which is absent *in vitro* may have mitigated effect of GCN5 inhibition, possibly via signaling bypass, while combination therapy that blocks more axes may have rendered shunting signaling unresponsive, given the relative short period of xenograft growth.

Given that CCNE1 is amplified in a variety of cancers, targeting tumors with this genotype is a hotspot of investigation. Rb can be phosphorylated to promote the progression of G1 when the biosynthetic activities are at a high rate, and the over-phosphorylated Rb will accelerate the transition from G1 to S phase [16]. Moreover, it is reported that the time point to entry S phase is dependent on cyclin E levels [17]. Cyclin E-CDK2 can also phosphorylate Smad3 and inhibit the transcriptional activity so as to shorten the cell cycle progression [18]. More importantly, cyclin E is observed to be overexpressed in many types of tumor cells, including breast, lung, bladder and colon cancer [19]. Based on those knowledge, we tested acetylation Rb as well and found that GCN5 or CDK2 knockdown reduced acetylation of Rb. Targeting CDK2 has thus far been the only targeting approach for CCNE1 amplified cancer. A recent screen on synthetic lethality also shows that amplification of CCNE1 is dependent on CDK2 activity [20]. Also, as we have now observed metabolic shift in Cyclin E1-driven OvCa, we are now performing a metabolic profile analysis to comprehensively pinpoint metabolic vulnerability following CDK2 and GCN5 monotherapy or combined. Those data may help overcome drug resistance to CDK2 inhibitors. To sum up, our study showed that targeting GCN5 could inhibit metabolic mediation of the CCNE1-CDK2 complex and combined inhibition with CDK2 blockade held promise for drug development.

## MATERIALS AND METHODS

### In silico analysis

The TCGA database of high-grade serous ovarian cancer were utilized and analyzed using the cBioPortal platform [21] and expressions of genes were studied using the TIMER platform [22, 23]. We queried samples with CCNE1 amplification for mRNA expression enrichment. Significantly enriched genes were analyzed using the NET-GE: NETwork-based Gene Enrichment system [24].

### Cell lines

Cyclin E1-driven ovarian cancer cell lines (OVCAR-3 and A2780) were selected from the COSMIC database (http://cancer.sanger.ac.uk/cancergenome/projects/cosmic/) and obtained from the cell bank of Chinese Academy of Science. Both cell lines were cultured in complete RPMI-1640 media supplemented with fetal bovine serum. Transcripts for shRNA construction targeting CCNE1, CDK2, GCN5 and PGC-1α (PPARGC1A) are selected from the RNAi Consortium (TRC, http://www.broadinstitute.org/rnai/public/). Two transcripts for each gene were selected for testing (CCNE1: TRCN0000045299 and TRCN0000045298; CDK2: Clone IDs TRCN0000196690 and TRCN0000321766; GCN5: TRCN0000364135 and TRCN0000018530; Clone IDs PPARGC1A: Clone IDs TRCN0000364085 and TRCN0000001165). Vectors with resistance to puromycin were constructed for both genes (pKLO.1-CDK2-Puro-CMV and pKLO.1-PPARGC1A-Puro-CMV) together with corresponding control vectors (pKLO.1-Puro-CMV) generating CDK2-knockdown (KD) and – wildtype (WT), GCN5-KD and –WT and PGC-1α-KD and –WT cells for both cell lines via non-lipofectamine Fugene transfection. 97μl of Optimedium were applied for balancing at room temperature for 1 h with 3μl of Fugene-6. Vectors were added therein with gentle mixing. After 20 min of incubation, the mixture was added to cells which were cultured overnight. Medium was replaced with complete medium supplemented with 1:5000 of puromycin and changed every 3 days until all clones were negative for CDK2 or PPARGC1A expression. Similar methods were used to generate control cells.

### Acetylation assay

To avoid degradation of PGC-1α and Retinoblastoma protein (Rb), cells were treated with RIPA buffer. Flag- PGC-1α and Rb were expressed in both cell lines. The Flag-tagged cells were then infected with GCN5. Forty-eight hours after infection, cells were treated with 50μM resveratrol or vehicle for 18h. The cells were then harvested, and cell extracts containing 200μg of protein were rotated with anti-Flag antibody at 4°C overnight. Agarose G beads were added and the samples were rotated at room temperature for 2h. The agarose beads were washed 4 times with PBS and protein was eluted from the beads with 5× SDS buffer. PGC-1α and Rb were measured with corresponding antibodies, and levels of acetylation were then assessed with an anti-acetyl lysine antibody.

### mRNA expression and western blotting

Total RNA was extracted with Trizol reagent and was converted to cDNA. Primers for CCNE1, G6PC, GCN5, PPARGC1A and CDK2 were designed using the PrimerBank (https://pga.mgh.harvard.edu/primerbank/) and corresponding primers were listed as follows: CCNE1 (forward, AAG GAG CGG GAC ACC ATG A; reverse, ACG GTC ACG TTT GCC TTC C), G6PC (forward, CTA CTA CAG CAA CAC TTC CGT G; reverse, GGT CGG CTT TAT CTT TCC CTG A), GCN5 (forward, CAG GGT GTG CTG AAC TTT GTG; reverse, TCC AGT AGT TAA GGC AGA GCA A), PPARGC1A (forward, TCT GAG TCT GTA TGG AGT GAC AT; reverse, CCA AGT CGT TCA CAT CTA GTT CA) and CDK2 (forward, GTA CCT CCC CTG GAT GAA GAT; reverse, CGA AAT CCG CTT GTT AGG GTC). The relative expression level of each gene was determined by the real-time quantitative PCR using SYBR Premix Ex Taq II (Takara, Tokyo, Japan). For western blotting, a standard protocol was followed. Total protein was first extracted and concertation was then determined. Proteins were then separated by SDS-PAGE on a 10% gel and subsequently transferred to a polyvinylidene difluoride membrane. Non-specific antigens were blocked using non-fat milk and corresponding primary antibodies were applied according to manufacturer’s recommended protocol and concentration as follows: anti-PGC-1α (Santa Cruz Biotechnology, 1:100), anti-CDK2 (ThermoFisher, 1:500), anti-GCN5 (ThermoFisher, 1:2000), anti-Cyclin E1 (ThermoFisher, 1:1000) and ß-catenin (Santa Cruz Biotechnology, 1:200). After corresponding secondary antibodies were applied, protein bands were then detected using chemiluminescence and autoradiography for densitometry detection.

### Flow cytometry

Cell cycle and apoptosis were detected using flow cytometry on a FASCanto System. For cell cycle analysis, cells were first rinsed and fixed with chilled ethanol. Cell cycle staining buffer was then applied and cells were processed on FASCanto. For apoptosis, cells were harvested and treated with Annexin V and PI. Apoptosis was designated as the sum of early and late apoptotic cells.

### Colorimetric assays

The glucose uptake assay, lactate assay and TCA cycle activity detection were all performed on a colorimetric basis using a plate reader. The AAT kit was used to quantify glucose uptake in media. Cells were harvested and treated as per manufacturer’s protocol. The NADPH was detected on a plate reader for absorbance OD at 570 - 610nm. The colorimetric L-lactate assay kit was used to quantify lactate secretion. A standard protocol of the manufacturer was followed. Lactate secretion of the cells was measured at a wavelength of 450 nm on a plate reader. The TCA activity was profiled by Succinate dehydrogenase (SDH) and the Succinate Dehydrogenase Activity Assay Kit was used.

### Transwell assays

Transwell assay was used to study cell migration and invasion. Briefly, cells were resuspended at the density of 1×10^6^/ml in 300μl of serum free medium and seeded in the upper chamber of Transwell without any coating for migration and with Matrigel coating for invasion. The lower chambers were then filled with 500 μl of complete medium and cells migrated through the membrane were stained with crystal violet and counted.

### Colony formation assays

Colony formation assay was used to profile anchorage-independent growth. Cells were seeded in in medium containing 10% FBS with 0.4% agarose, which was layered on the top of 0.6% agar in medium supplemented with 20% FBS on 60-mm plates. After 2 weeks of culture at 37 °C, plates were stained with 0.005% of crystal violet for 1 h. Colonies were counted microscopically and the relative colony numbers were measured.

### Xenograft mouse model

OvCa xenograft mouse models using OVCAR-3 and A2780 cells has been well-stablished by our group. Four groups for each cell lines were set and Dinaciclib was administrated intraperitoneally at the dose of 50 mg/kg, whilst shGCN5 and control plasmid were delivered to both cell lines before implantation. All mice were sacrificed on Day 35 and tumors were extracted. Tumor size was calculated with the formula, Length * Width^2^ * 0.5236.

### Statistical analysis

Comparisons between groups were analyzed with the 2-tailed Student’s t-test. The P value of 0.05 was accepted as statistically significant.

## References

[1] Bray F, Ferlay J, Soerjomataram I, Siegel RL, Torre LA, Jemal A. Global cancer statistics 2018: GLOBOCAN estimates of incidence and mortality worldwide for 36 cancers in 185 countries. CA Cancer J Clin. 2018; 68:394–424. 10.3322/caac.21492 30207593

[2] Seidman JD, Horkayne-Szakaly I, Haiba M, Boice CR, Kurman RJ, Ronnett BM. The histologic type and stage distribution of ovarian carcinomas of surface epithelial origin. Int J Gynecol Pathol. 2004; 23:41–44. 10.1097/01.pgp.0000101080.35393.16 14668549

[3] Wang K, Li D, Sun L. High levels of EGFR expression in tumor stroma are associated with aggressive clinical features in epithelial ovarian cancer. Onco Targets Ther. 2016; 9:377–86. 10.2147/OTT.S96309 26855586 PMC4727521

[4] Cancer Genome Atlas Research Network. Integrated genomic analyses of ovarian carcinoma. Nature. 2011; 474:609–15. 10.1038/nature10166 21720365 PMC3163504

[5] Noske A, Henricksen LA, LaFleur B, Zimmermann AK, Tubbs A, Singh S, Storz M, Fink D, Moch H. Characterization of the 19q12 amplification including CCNE1 and URI in different epithelial ovarian cancer subtypes. Exp Mol Pathol. 2015; 98:47–54. 10.1016/j.yexmp.2014.12.004 25527175

[6] Taylor-Harding B, Aspuria PJ, Agadjanian H, Cheon DJ, Mizuno T, Greenberg D, Allen JR, Spurka L, Funari V, Spiteri E, Wang Q, Orsulic S, Walsh C, et al. Cyclin E1 and RTK/RAS signaling drive CDK inhibitor resistance via activation of E2F and ETS. Oncotarget. 2015; 6:696–714. 10.18632/oncotarget.2673 25557169 PMC4359249

[7] Guo T, Gu C, Chen X, Kang Y, Li B, Xu C. Inhibition of succinate dehydrogenase sensitizes cyclin E-driven ovarian cancer to CDK inhibition. Biofactors. 2016; 42:171–78. 26826064 10.1002/biof.1257

[8] Guo T, Chen T, Gu C, Li B, Xu C. Genetic and molecular analyses reveal G6PC as a key element connecting glucose metabolism and cell cycle control in ovarian cancer. Tumour Biol. 2015; 36:7649–58. 10.1007/s13277-015-3463-6 25926381

[9] Sakai M, Matsumoto M, Tujimura T, Yongheng C, Noguchi T, Inagaki K, Inoue H, Hosooka T, Takazawa K, Kido Y, Yasuda K, Hiramatsu R, Matsuki Y, Kasuga M. CITED2 links hormonal signaling to PGC-1α acetylation in the regulation of gluconeogenesis. Nat Med. 2012; 18:612–17. 10.1038/nm.2691 22426420

[10] Sun Y, Yang C, Chen J, Song X, Li Z, Duan M, Li J, Hu X, Wu K, Yan G, Yang C, Liu J, Tan W, Ye M. Overexpression of WDR79 in non-small cell lung cancer is linked to tumour progression. J Cell Mol Med. 2016; 20:698–709. 10.1111/jcmm.12759 26849396 PMC5125931

[11] Marone M, Scambia G, Giannitelli C, Ferrandina G, Masciullo V, Bellacosa A, Benedetti-Panici P, Mancuso S. Analysis of cyclin E and CDK2 in ovarian cancer: gene amplification and RNA overexpression. Int J Cancer. 1998; 75:34–39. 10.1002/(SICI)1097-0215(19980105)75:1<34::AID-IJC6>3.0.CO;2-2 9426687

[12] Lee Y, Dominy JE, Choi YJ, Jurczak M, Tolliday N, Camporez JP, Chim H, Lim JH, Ruan HB, Yang X, Vazquez F, Sicinski P, Shulman GI, Puigserver P. Cyclin D1-Cdk4 controls glucose metabolism independently of cell cycle progression. Nature. 2014; 510:547–51. 10.1038/nature13267 24870244 PMC4076706

[13] Pal A, Potjer TP, Thomsen SK, Ng HJ, Barrett A, Scharfmann R, James TJ, Bishop DT, Karpe F, Godsland IF, Vasen HF, Newton-Bishop J, Pijl H, et al. Loss-of-Function Mutations in the Cell-Cycle Control Gene CDKN2A Impact on Glucose Homeostasis in Humans. Diabetes. 2016; 65:527–33. 10.2337/db15-0602 26542317 PMC4724950

[14] Majaz S, Tong Z, Peng K, Wang W, Ren W, Li M, Liu K, Mo P, Li W, Yu C. Histone acetyl transferase GCN5 promotes human hepatocellular carcinoma progression by enhancing AIB1 expression. Cell Biosci. 2016; 6:47. 10.1186/s13578-016-0114-6 27486509 PMC4969657

[15] Holmlund T, Lindberg MJ, Grander D, Wallberg AE. GCN5 acetylates and regulates the stability of the oncoprotein E2A-PBX1 in acute lymphoblastic leukemia. Leukemia. 2013; 27:578–85. 10.1038/leu.2012.265 23044487

[16] Hinds PW, Mittnacht S, Dulic V, Arnold A, Reed SI, Weinberg RA. Regulation of retinoblastoma protein functions by ectopic expression of human cyclins. Cell. 1992; 70:993–1006. 10.1016/0092-8674(92)90249-C 1388095

[17] Dulić V, Lees E, Reed SI. Association of human cyclin E with a periodic G1-S phase protein kinase. Science. 1992; 257:1958–61. 10.1126/science.1329201 1329201

[18] Cooley A, Zelivianski S, Jeruss JS. Impact of cyclin E overexpression on Smad3 activity in breast cancer cell lines. Cell Cycle. 2010; 9:4900–07. 10.4161/cc.9.24.14158 21150326 PMC3047813

[19] Donnellan R, Chetty R. Cyclin E in human cancers. FASEB J. 1999; 13:773–80. 10.1096/fasebj.13.8.773 10224221

[20] McDonald ER 3rd, de Weck A, Schlabach MR, Billy E, Mavrakis KJ, Hoffman GR, Belur D, Castelletti D, Frias E, Gampa K, Golji J, Kao I, Li L. Project DRIVE: A Compendium of Cancer Dependencies and Synthetic Lethal Relationships Uncovered by Large-Scale, Deep RNAi Screening. Cell. 2017; 170:577–592. 10.1016/j.cell.2017.07.005 28753431

[21] Gao J, Aksoy BA, Dogrusoz U, Dresdner G, Gross B, Sumer SO, Sun Y, Jacobsen A, Sinha R, Larsson E, Cerami E, Sander C, Schultz N. Integrative analysis of complex cancer genomics and clinical profiles using the cBioPortal. Sci Signal. 2013; 6:pl1. 10.1126/scisignal.2004088 23550210 PMC4160307

[22] Li T, Fan J, Wang B, Traugh N, Chen Q, Liu JS, Li B, Liu XS. TIMER: A Web Server for Comprehensive Analysis of Tumor-Infiltrating Immune Cells. Cancer Res. 2017; 77:e108–10. 10.1158/0008-5472.CAN-17-0307 29092952 PMC6042652

[23] Li B, Severson E, Pignon JC, Zhao H, Li T, Novak J, Jiang P, Shen H, Aster JC, Rodig S, Signoretti S, Liu JS, Liu XS. Comprehensive analyses of tumor immunity: implications for cancer immunotherapy. Genome Biol. 2016; 17:174. 10.1186/s13059-016-1028-7 27549193 PMC4993001

[24] Di Lena P, Martelli PL, Fariselli P, Casadio R. NET-GE: a novel NETwork-based Gene Enrichment for detecting biological processes associated to Mendelian diseases. BMC Genomics. 2015 (Suppl 8); 16:S6. 10.1186/1471-2164-16-S8-S6 26110971 PMC4480278

