# Correction for: GCN-5/PGC-1α signaling is activated and associated with metabolism in cyclin E1-driven ovarian cancer

**DOI:** 10.18632/aging.205948

**Published:** 2024-05-31

**Authors:** Ting Guo, Bin Li, Chao Gu, Xiuying Chen, Mengxin Han, Xiaocheng Liu, Congjian Xu

**Affiliations:** 1 Department of Gynecology, Obstetrics and Gynecology Hospital of Fudan University, Shanghai 200011, PR China

**Keywords:** high grade serous ovarian cancer, CCNE1, glucose metabolism

**This article has been corrected:** The authors found that in four instances, they had inadvertently used the incorrect image when assembling **Figure 3**. To correct the figure, they replaced the incorrect Cyclin E1 image in Figure 3A, CDK2 and ß-actin images in Figure 3B, and PGC-1α image in Figure 3C with the correct images from the original experiments. The authors state that this correction has no impact on the main conclusion. The authors apologize for any inconvenience to the readers.

New **Figure 3** is presented below.

**Figure 3:**
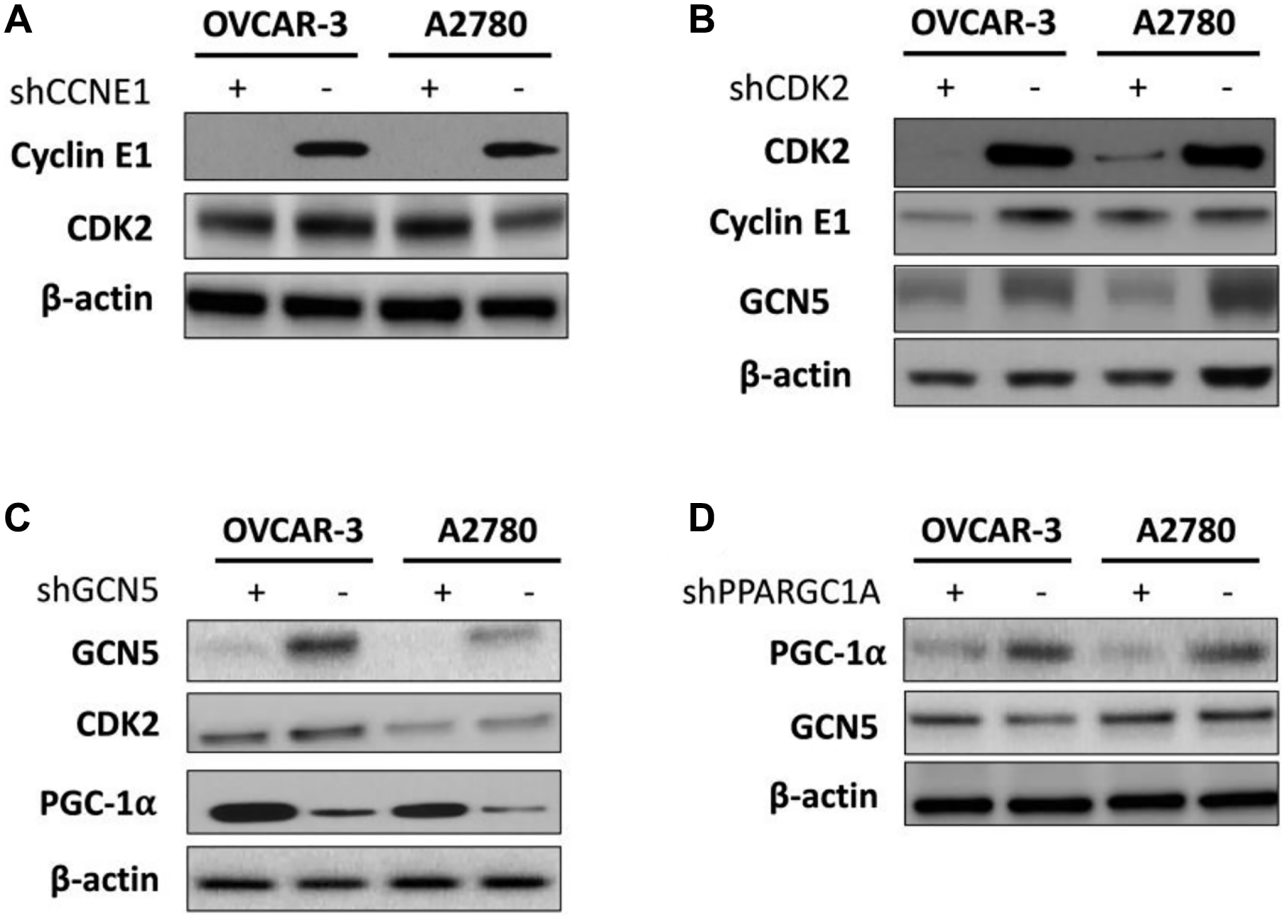
Shown are hierarchy of regulation demonstrating impact of (**A**) silencing of CCNE1 on CDK2; (**B**) silencing of CDK2 on Cyclin E1 and GCN5; (**C**) silencing of GNG5 on CDK2 and PGC-1α; and (**D**) silencing of PGC-1α onGCN5.

